# Extracorporeal membrane oxygenation rescue for severe pneumocystis pneumonia with the Macklin effect: a case report

**DOI:** 10.1186/s12879-022-07550-9

**Published:** 2022-06-27

**Authors:** Guoqing Huang, Liping Zhou, Ning Yang, Ping Wu, Xiaoye Mo

**Affiliations:** Department of Emergency, Xiangya Hospital, Central South University, Changsha, Hunan People’s Republic of China

**Keywords:** Extracorporeal membrane oxygenation, *Pneumocystis jirovecii* pneumonia, Macklin effect, Case report

## Abstract

**Background:**

*Pneumocystis jirovecii* pneumonia (PJP) in an immunocompromised host is often associated with the Macklin effect, which can progress to spontaneous pneumomediastinum (SPM), subcutaneous emphysema (SCE), and pneumothorax (PNX). Diagnosing the causative organism of these conditions in non-HIV infected patients and treating hypoxemia while preventing further lung damage can be challenging. This study examines the case of a non-HIV infected male with SPM, SCE, and PNX secondary to severe *Pneumocystis jirovecii* (PJ) infection.

**Case presentation:**

A 53-year-old male with pure red cell aplasia (PRCA) was admitted with fever, dry cough, and shortness of breath. His respiratory function progressively deteriorated due to the development of SPM, SCE, and PNX, eventually requiring endotracheal intubation and invasive ventilation. As a result of high pressure in his airways occasioned by lung recruitment maneuvers, his pulmonary parameters worsened, necessitating veno-venous (VV) extracorporeal membrane oxygenation (ECMO) therapy. The early initiation of VV-ECMO facilitated ultra-protective lung ventilation and prevented the progression of SPM, SCE, and PNX. Traditional diagnostic assays were unrevealing, whereupon the patient resorted to the metagenomic next-generation sequencing technology for uncovering potential pathogens. Consequently, we detected a significantly higher infection by PJ in the patient’s bronchoscopy lavage fluid. Finally, the patient was successfully treated with appropriate antimicrobials and was decannulated after nine days of ECMO support.

**Conclusions:**

SPM, SCE, and PNX are rare clinical manifestations of PJP. However, they can be considered as poor prognostic factors of the infection. Physicians should, therefore, be alert to the possibility of PJP in immunocompromised patients.

## Background

*Pneumocystis jirovecii* pneumonia (PJP) is a severe and potentially fatal opportunistic infection in immunosuppressed patients [1]. Common clinical signs of PJP include fever, chest pain, dry cough, and dyspnea [2]. Severe PJP may be associated with the Macklin effect, clinically presenting as spontaneous pneumomediastinum (SPM), subcutaneous emphysema (SCE), and pneumothorax (PNX) [3, 4]. Sherman et al. [5] and Villalona-Calero et al. [6] were the first to report that PNX and SPM complicated PJP in patients with AIDS. The Macklin effect, which is often the precursor of SPM in HIV patients, is relatively uncommon in patients without HIV—with an incidence rate of 0.4–4% [7]. In this report, we reviewed the case of a 53-year-old man with pure red cell aplasia (PRCA) who developed a life-threatening complication of PJP due to severe respiratory distress syndrome. Multiple studies have established that non-HIV infected patients with SPM, SCE, and PNX are more prone to developing severe symptoms and complications [8–10].

### Case presentation

In October 2019, a 53-year-old man with PRCA visited the emergency department of Xiangya Hospital, Changsha, China. His family history and psycho-social history were not special. The patient had experienced acute dyspnea for 5 days, accompanied by fever and cough without sputum. To treat PRCA, the patient had taken oral prednisone irregularly before the 5-day period. On admission, he was febrile (37.9 °C), tachypneic (30 breaths/min), and had a pulse oxygen saturation (SpO2) value of 80%. His blood pressure and pulse rate were within normal ranges. The breath sounds of both lungs were clear, without dry or wet rale. Furthermore, the heart boundary was normal, and there were no heart murmurs.

**Fig. 1:**
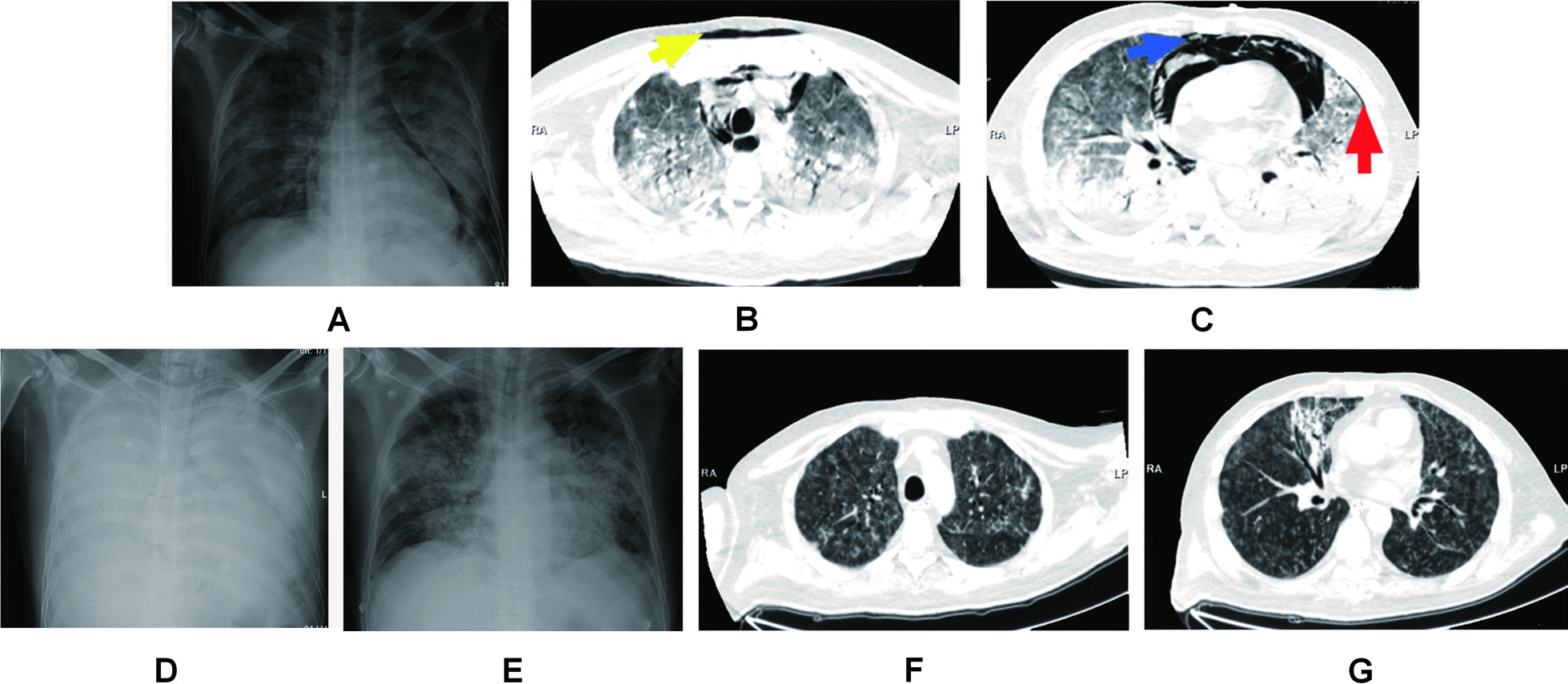
Chest X-ray and CT scans of patient. **A** On admission: bilateral middle and lower zone air-space opacities; hyperlucency at the edge of mediastinum structures, suggestive of pneumomediastinum. **B** The axial CT scans at presentation: It shows diffuse bilateral opacities in bilateral lung, a pneumomediastinum, as well as minor subcutaneous emphysema in front of the sternum (yellow arrow). **C** The CT scan of bilateral middle and lower zones: it shows diffuse ground-glass opacity and extensive basal consolidation, a large pneumomediastinum as the air in the anterior and superior mediastinum (blue arrow) and a small left pneumothorax (red arrow). **D** Post-ECMO cannulation: X-ray of two-sided “White Lung” after ECMO performance. **E** Decannulation after 9 days of ECMO support: disappearance of pneumomediastinum and absorption of ground-glass opacities. **F** The corresponding axial CT scans after 29 days: it shows a reduction of the pulmonary inflammatory process and pneumomediastinum resorption. **G** The corresponding axial CT scans of **C** after 29 days: a marked reduction of pneumomediastinum and a complete resolution of pneumothorax is evident in the corresponding scan. A small glass opacity was shown in the medial segment of the right middle lobe

The main abnormalities in laboratory findings on admission were as follows: Total leukocyte, 15.2 × 10^9^/L (normal reference, NR: 4.0–10.0 × 10^9^/L); neutrophils, 14.6 × 10^9^/L (NR: 1.8–6.3 × 10^9^/L); lymphocytes, 0.3 × 10^9^/L (NR: 1.1–3.2 × 10^9^/L); albumin, 17.5 g/L (NR: 40.0–55.0 × g/L); lactate dehydrogenase, 656.0 U/L (NR: 120.0–250.0 U/L); glucose, 18.92 mmol/L (NR: 3.90–6.10 mmol/L); D-dimer, 0.55 mg/L (NR: 0–0.5 mg/L); procalcitonin (PCT), 0.61 ng/mL (NR: 0–0.046 ng/mL); C-reactive protein, 116.14 mg/L (NR: 0–8.00 mg/L); and erythrocyte sedimentation rate, 120 mm/h (NR: 0–21 mm/h). The liver and kidney functions were normal. Also, the HIV test was negative. We initiated blood culture and sputum culture upon admission. Because of a low SpO2 under nasal oxygen and poor consciousness, the patient was transferred to an emergency intensive care unit (EICU). He received a series of respiratory supportive maneuvers, such as high flow oxygenation for one hour and non-invasive ventilation for two hours. Then, initial mobile chest X-ray revealed air-space opacities in the bilateral middle and lower zones and hyperlucency at the edge of mediastinum structures, which is suggestive of pneumomediastinum (Fig. 1A). In addition, a chest CT scan was performed, which showed diffuse, patchy, ground-glass opacities in bilateral lungs, along with a large pneumomediastinum, small SCE in front of the sternum, and a small left PNX (Fig. 1B, C). However, the patient’s respiratory status did not improve with the respiratory maneuvers. As a result, he was placed on endotracheal intubation, starting with invasive ventilation in emergency scenarios. The ventilatory protocol included a pressure control rate of 16 breaths/min, a fraction of inspired oxygen (FiO_2_) of 100%, a positive end-expiratory pressure (PEEP) of 10 cmH_2_O, and a driving pressure of 20 cmH_2_O. At that time, the patient received an anti-viral treatment, with 75 mg of oseltamivir twice daily, accompanied by 4.5 g of piperacillin-tazobactam every 8 h. This broad-spectrum antibiotic therapy because of his compromised immune system resulting from a history of prednisone medication.

**Fig. 2:**
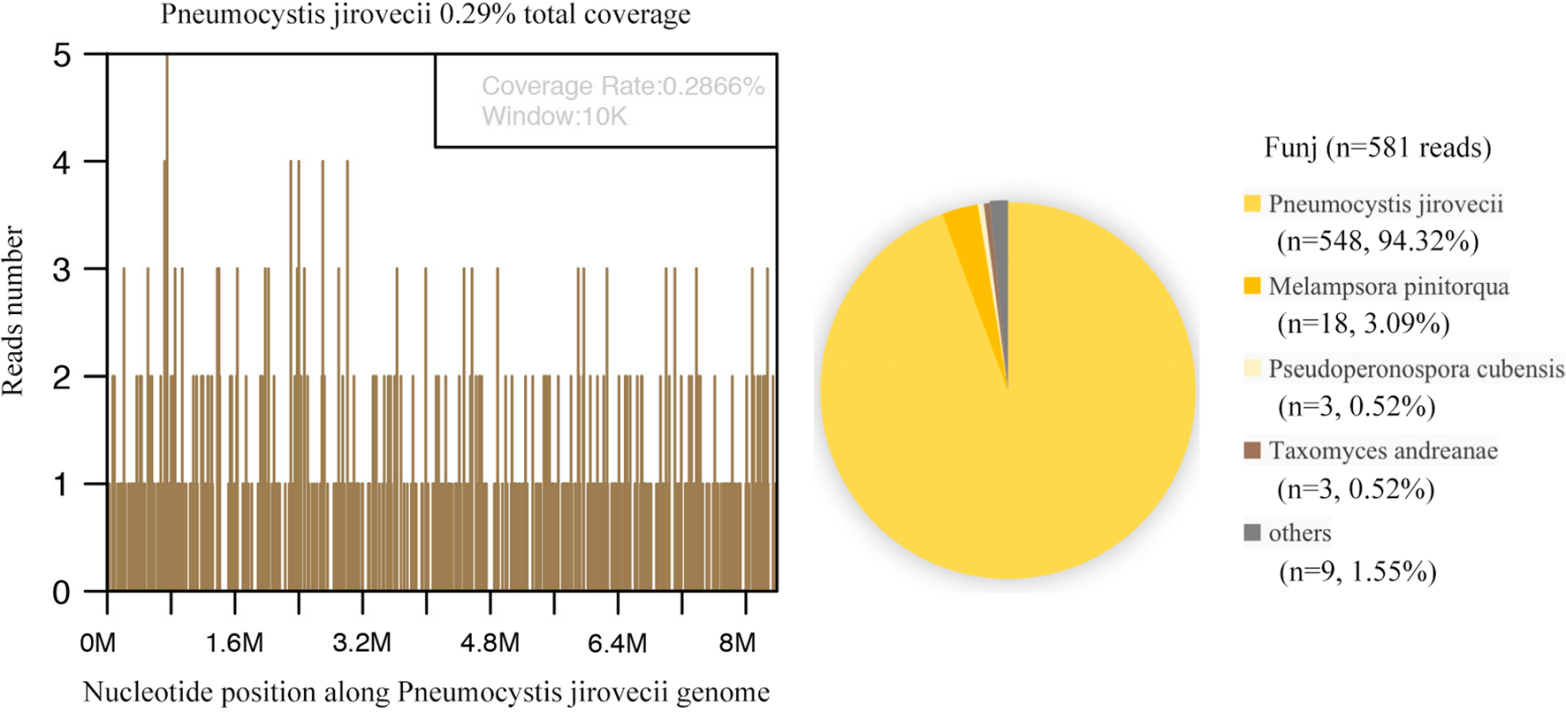
mNGS results of pathogen identification. In this case, 94.32% of funj reads corresponded to *Pneumocystis jirovecii*, with a coverage of 0.29%. mNGS: the metagenomic next-generation sequencing

**Fig. 3:**
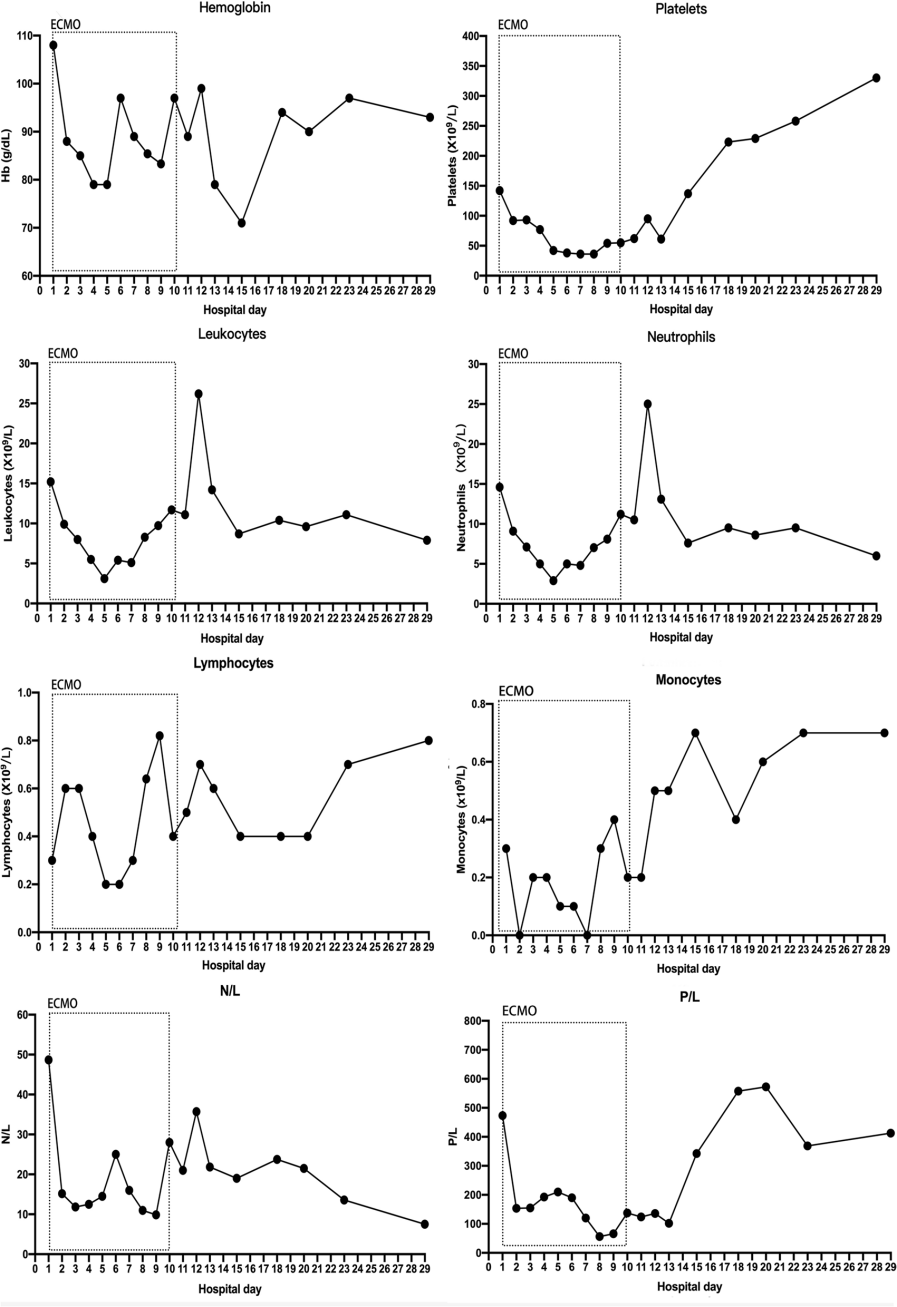
Evolution of blood count markers during hospitalization. Hemogram was evaluated throughout the hospital stay. Hemoglobin, total leukocytes, neutrophil count, lymphocytes, monocytes, platelets, neutrophil-to-lymphocyte (N/L) ratios, and platelet-to-lymphocyte (P/L) were represented with a connected scatterplot

**Fig. 4:**
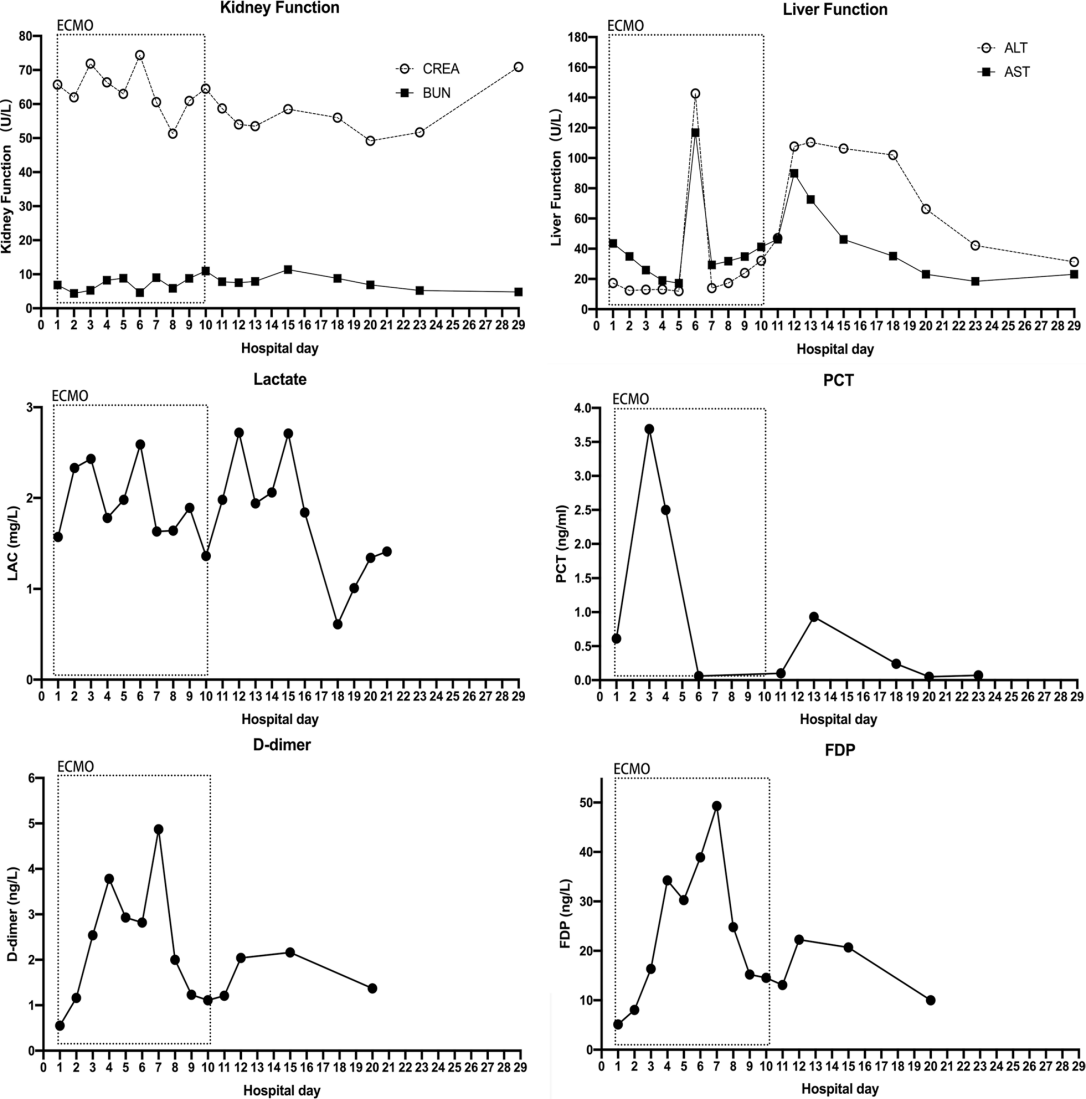
Evolution of biochemical markers and biochemical tests during hospitalization. AST and ALT were combined to represent liver function. Urea and creatinine were combined to represent renal function. Together with LDH, PCT, D-dimer and FDP, these connected scatterplots show the physiological evolution of the patient during hospital day. *AST* aspartate aminotransferase, *ALT* alanine aminotransferase, *PCT* procalcitonin, *LDH* lactate dehydrogenase, *FDP* fibrin degradation products

The patient’s hypoxemia (PaO_2_ < 50 mmHg) continued into the second day of hospitalization. The medical professionals were unable to perform prone positioning and lung recruitment on the patient because these therapeutic maneuvers would worsen his hypoxemia and facilitate the progression of the Macklin effect. Emergency veno-venous (VV) extracorporeal membrane oxygenation (ECMO) was thus initiated as an ultra-protective lung ventilation strategy. The general ECMO settings were as follows: flow rate, 3.5–5 L/min; sweep, 2.0 L/min; and FiO_2_, 100%. Ventilator settings were adjusted after initiating VV-ECMO, with decreases in FiO_2_ from 100 to 40% and a PEEP from 10 cm H_2_O to 5 cm H2O. A chest X-ray of two-sided “white lung” after ECMO performance was shown (Fig. 1D). The patient’s blood culture and sputum culture came out negative on the third day of hospitalization, confirming no bacterial infection. The G-test value was therefore elevated (600 pg/mL, NR: < 70). The patient tested negative for common viruses, necessitating the suspension of oseltamivir administration. Since traditional diagnostic assays were unrevealing, the patient resorted to the metagenomic next-generation sequencing technology for uncovering potential pathogens. Therefore, DNA sequencing was performed based on the BGISEQ-100 platform (BGI-Tianjin, Tianjin, China). High-quality sequencing data were generated after filtering out low-quality, low-complexity, and shorter reads. Using Burrows–Wheeler Alignment (BWA, http://bio-bwa.sourceforge.net/), the data mapped to the human reference genome were excluded. The remaining data were aligned to the Microbial Genome Database from NCBI, including bacteria, viruses, fungi, and protozoa (http://ftp.ncbi.nlm.nih.gov/genomes/). Finally, the mapped data were processed by removing duplicate reads for advanced data analysis. As a result, we detected a significantly higher infection by PJ in the patient’s bronchoscopy lavage fluid and confirmed the diagnosis of PJP (Fig. 2). Then, the patient was started on trimethoprim/sulfamethoxazole (SMZ-TMP) at a dose of 0.96 g every 6 h. On day 6 of hospitalization, the patient’s condition evolved with an improvement of the fever. Figures 3 and 4 showed the changes in laboratory findings following the VV-ECMO procedure. Except hemogram evaluated throughout the hospital stay, Fig. 3 also showed the alterations of neutrophil-lymphocyte count ratio (N/L) and platelet-lymphocyte count ratio (P/L), which were recognized as useful predictors of disease severity and worse prognosis. Figure 4 showed the kidney and liver functions of this patient. A temperate hepatic dysfunction on day 6 of hospitalization caused by PJ was relieved soon after protective treatment, without kidney injury. Serum lactate levels were increased at the early stage of PJP, and PCT as a marker of infection decreased gradually after the administration of SMZ-TMP.

Meanwhile, plasma D-dimer and fibrin degradation products, as two promising biomarkers to predict perioperative fibrinolysis and bleeding, increased during ECMO performance and decreased after ECMO withdrawal. As of day 9 of hospitalization, the patient had been on antimicrobial therapy for 6 days and showed progressive improvement in respiratory failure, with the disappearance of SPM and absorption of ground-glass opacities in X-ray (Fig. 1E). The ECMO cannulas were removed on day 10 of hospitalization. However, the patient needed prolonged ventilator support and tracheostomy. A repeat CT scan of the chest also showed resorption and disappearance of SPM, SCE, and PNX (Fig. 1F, G). On day 23 of hospitalization, the patient regained consciousness, with his invasive ventilation and tracheal tube removed successfully. No adverse or unanticipated events occurred. After 29 days of hospitalization, he was discharged. With two years of follow-up, the patient showed a good prognosis without recurrence. The patient could understand the duration and efficacy of the treatment.

## Discussion and conclusions

We successfully used VV-ECMO in a non-HIV patient with severe PJP infection. The patient had suffered fatal respiratory failure accompanied by the Macklin effect, which progressed to SPM, SCE, and PNX. VV-ECMO was performed as a safe and effective rescue therapy to correct the aggravating hypoxemia and prevent other clinical complications. With the use of SMZ-TMP as a treatment, the patient’s condition improved, and the patient was discharged after 29 days. The discharge followed the successive removal of the patient from ECMO, invasive ventilation, and tracheal tube.

The Macklin effect often occurs in patients with pneumonia who develop acute respiratory distress syndrome (ARDS) and need invasive mechanical ventilation with high PEEP and driving pressure [3, 11]. Empirical evidence shows that SPM, SCE, and PNX rarely occur in patients without invasive mechanical ventilation [11, 12]. The exact pathophysiology involved in these complications is thought to be related to diffuse alveolar rupture. The pressure gradient between the alveoli and surrounding tissues increases once the pressure in the respiratory tract goes up, leading to the rupture of alveoli and consequent formation of interstitial emphysema. When the average pressure in the mediastinum is lower than that in the surrounding pulmonary parenchyma, the gas enters the mediastinum. The Macklin effect involves a three-step pathophysiologic process: blunt traumatic alveolar rupturing, air dissection along bronchovascular sheaths, and spreading of this blunt pulmonary interstitial emphysema into the mediastinum [3, 13]. Research suggests that pneumonia caused by several specific pathogens can trigger the Macklin effect. In 2004, a study on severe acute respiratory syndrome (SARS) patients in Hong Kong reported an 11.6% incidence rate of SPM in infected persons [14]. Udupa et al. [15] reported three cases of SCE and pneumomediastinum in children with influenza A virus subtype H1N1. Recent reports documented SPM, SCE, and PNX occurring in COVID-19 patients [11–13, 16–19]. Often, these patients require high PEEP, prone positioning, and frequent bronchoalveolar lavage. These procedures increase the intrathoracic pressure and barotrauma.

In the absence of such viruses, PJ rarely causes the Macklin effect. Yee et al. [20] reported a 31-year-old man with PJP suffered from respiratory failure caused by pneumomediastinum and bilateral PNX. The clinical manifestations and radiologic abnormalities of PJP are nonspecific, making it challenging to differentiate PJP from other opportunistic pulmonary infections. It is, therefore, common to miss the diagnosis of PJP in a non-HIV-infected patient. In the case presented in this study, PJ was not considered a possible infection, and so the patient did not receive early antibiotics. Such delays in administering the correct treatment result from a delay in pathogen detection [21]. Although there is no direct relationship between PJ infection and the occurrence of the Macklin effect, some researchers believe that the event of SPM, SCE, and PNX could be considered a poor prognostic factor.

SPM, SCE, and PNX hinder the upregulation of invasive ventilator pressure and the execution of therapeutic maneuvers such as prone position ventilation [22]. The wrong implementation of endotracheal intubation may potentiate damage to the tracheal wall, worsening respiratory failure [7]. For critical patients with severe respiratory failure, the Macklin effect complicates treatment. The treatment procedure VV-ECMO was initiated to solve this problem. VV-ECMO is used extensively in cardiothoracic surgery, both for hemodynamic and respiratory support [22]. It has proven to be an effective ultra-lung ventilation tool in maintaining adequate oxygenation. To ensure optimal oxygenation with VV-ECMO, the ventilator parameters can be downregulated to a safe level to prevent further alveoli injury. The use of VV-ECMO allows time for the antibiotics to show effect and for pulmonary parenchyma to heal. Antonacci et al. [23] reported using ECMO to manage a patient with SPM, SCE, and PNX during tracheal surgery. We recommend that ECMO be initiated early to rescue severe pneumonia with the Macklin effect.

In conclusion, delays in initiating antimicrobial therapy for PJP is common among non-HIV-infected patients, even among those with access to modern medical care. This delay often leads to a worse outcome. Thus, quick and sensitive diagnostic assays to confirm or rule out PJP are of urgent importance. SPM, SCE, and PNX may be a series of indicators for a poor prognosis and a more severe condition. Physicians should be alert for PJP in severe pneumonia associated with SPM, SCE, and PNX. This case report highlighted the role of VV-ECMO in successfully managing this critical situation. VV-ECMO allows ultra-protective lung ventilation, thus preventing further lung injury.

## Data Availability

The data that included in this case are available from the corresponding author upon reasonable request.

## Abbreviations

ARDS: Acute respiratory syndrome
BWA: Burrows–Wheeler alignment
CRP: C-reactive protein
EICU: Emergency intensive care unit
ESR: Erythrocyte sedimentation rate
FiO_2_: Fraction of inspired oxygen
LDH: Lactate dehydrogenase
mNGS: Metagenomic next-generation sequencing
NR: Normal reference
N/L: Neutrophil–lymphocyte count ratio
PCT: Procalcitonin
PEEP: Positive end-expiratory pressure
PJ: *Pneumocystis jirovecii*
PJP: *Pneumocystis jirovecii* pneumonia
PNX: Pneumothorax
PRCA: Pure red cell aplasia
P/L: Platelet–lymphocyte count ratio
SCE: Subcutaneous emphysema
SMZ-TMP: Trimethoprim/sulfamethoxazole
SPM: Spontaneous pneumomediastinum
SpO_2_: Pulse oxygen saturation
VV-ECMO: Veno-venous extracorporeal membrane oxygenation
